# Rapid Changes in the Light/Dark Cycle Disrupt Memory of Conditioned Fear in Mice

**DOI:** 10.1371/journal.pone.0012546

**Published:** 2010-09-02

**Authors:** Dawn H. Loh, Juliana Navarro, Arkady Hagopian, Louisa M. Wang, Tom Deboer, Christopher S. Colwell

**Affiliations:** 1 Department of Psychiatry and Biobehavioral Sciences, University of California Los Angeles, Los Angeles, California, United States of America; 2 Department of Molecular Cell Biology, Leiden University Medical Center, Leiden, The Netherlands; University of Alberta, Canada

## Abstract

**Background:**

Circadian rhythms govern many aspects of physiology and behavior including cognitive processes. Components of neural circuits involved in learning and memory, e.g., the amygdala and the hippocampus, exhibit circadian rhythms in gene expression and signaling pathways. The functional significance of these rhythms is still not understood. In the present study, we sought to determine the impact of transiently disrupting the circadian system by shifting the light/dark (LD) cycle. Such “jet lag” treatments alter daily rhythms of gene expression that underlie circadian oscillations as well as disrupt the synchrony between the multiple oscillators found within the body.

**Methodology/Principal Findings:**

We subjected adult male C57Bl/6 mice to a contextual fear conditioning protocol either before or after acute phase shifts of the LD cycle. As part of this study, we examined the impact of phase advances and phase delays, and the effects of different magnitudes of phase shifts. Under all conditions tested, we found that recall of fear conditioned behavior was specifically affected by the jet lag. We found that phase shifts potentiated the stress-evoked corticosterone response without altering baseline levels of this hormone. The jet lag treatment did not result in overall sleep deprivation, but altered the temporal distribution of sleep. Finally, we found that prior experience of jet lag helps to compensate for the reduced recall due to acute phase shifts.

**Conclusions/Significance:**

Acute changes to the LD cycle affect the recall of fear-conditioned behavior. This suggests that a synchronized circadian system may be broadly important for normal cognition and that the consolidation of memories may be particularly sensitive to disruptions of circadian timing.

## Introduction

Daily rhythms in behavior and physiology are found in almost all organisms. The ability to synchronize ones physiology to anticipate environmental changes is thought to be the driving force behind the evolution of a network of circadian oscillators that adapt and respond, and yet have the ability to “keep time” in absence of any external cues. In mammals, the most critical of these environmental cues is light. The light signal is detected, in part, by photosensitive cells in the retinal ganglion layer [1], and is integrated by the master pacemaker in the hypothalamus: the suprachiasmatic nucleus (SCN) [2]. The SCN in turn coordinates a network of circadian oscillators that are found throughout the body [3], [4]. Within the brain, components of the circuits involved in learning and memory demonstrate rhythms in gene expression, including the amygdala [5] and the hippocampus [6], [7]. Importantly, these rhythms are autonomous as they continue in hippocampal slices in culture [8]. We hypothesize that these independent circadian oscillators in the learning and memory circuits are critical for providing a temporal structure to cognitive functions.

There are several lines of evidence that the circadian system can influence cognitive functions, especially memory. Perhaps the most important is the observation that peak performance in the recall of a number of behavioral tasks shows a diurnal [9]–[12] as well as a circadian variation [13], [14], [15]. The evidence for circadian regulation of gene expression [5], [7], [8], signaling pathways [8], [16], [17], and synaptic plasticity [18], [19], [20] in brain regions involved in learning and memory (e.g. the hippocampus) provide the mechanistic underpinnings to explain this temporal regulation. Mutations that have an effect on the circadian molecular timing loop [8], [21]–[25] and cellular communication within the SCN clock [26] affect the recall of learned behavior, as do mutations in the rhythmically regulated cAMP/ERK/CREB pathway (e.g., [17]). Similarly, environmental manipulations that disrupt circadian rhythms without genetic mutations also disrupt memory in different tasks [27]–[30]. For example, previous work has provided clear evidence that chronic phase shifts of the light/dark (LD) cycle interfere with memory [31], [32], [33]. Less work has examined the impact of single alterations in the timing of the LD cycle [34], even though these phase shifts disrupt the rhythms in clock gene expression within the SCN [35], [36] and between the SCN and peripheral oscillators [37].

Therefore, we performed a series of experiments to test the hypothesis that acutely altering the LD cycle can affect the acquisition and recall of contextual fear conditioning in mice. By subjecting mice to this experimental “jet lag” on the day before or after training, we addressed the importance of entrainment to the LD cycle on acquisition and recall. Further experiments explored the degree that recall was affected by the duration and direction of phase shifts. We measured the impact of these phase shifts on the stress response and sleep in the mice. Finally, we also explored the possibility that prior experience of phase shifts could compensate for the negative effect of acute phase shifts on recall.

## Results

### Does an acute phase shift prior to fear conditioning affect acquisition or recall?

**Figure 1:**
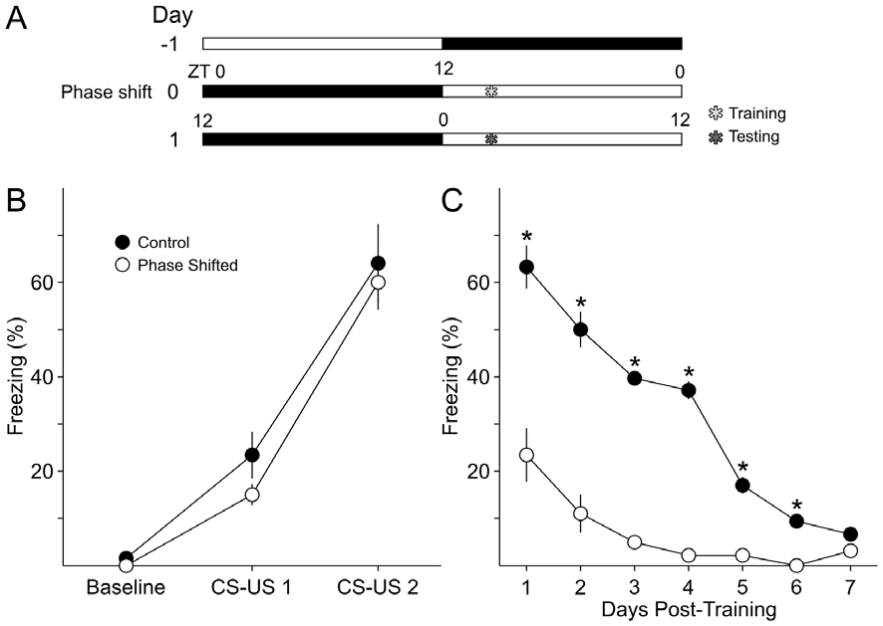
Phase shift prior to training reduced recall, but not acquisition, of contextual fear-conditioned behavior. **A**) Schematic illustration of the experimental design. In this and all subsequent experiments, adult male mice were entrained to a 12∶12 LD cycle for at least 2 weeks. One group (*n* = 6) was subjected to a 12 hr extension of the dark phase on Day -1 to cause a phase inversion by Day 0. On Day 0, both the phase shifted and the control group (*n* = 8) were trained at ZT 3. 24 hr after training, both groups of mice were returned to the same context for testing of the recall in once a day. **B**) Acquisition of the conditioned fear behavior was not altered by the phase shift. Freezing in response to CS-US 1 and CS-US 2 was not different between the control and phase shifted groups. **C**) Recall of the conditioned fear was dramatically reduced by the phase shift. The phase shifted group displayed significantly reduced freezing compared to the control group upon testing. In this and subsequent experiments, two way repeated measures analysis of variance (2RM ANOVA) followed by Bonferroni's pairwise multiple comparison *t*-tests were used to assess differences between the cohorts. The criterion level for significance was set at *P*<0.05, and the “*” symbol indicates significant differences between control and phase-shifted groups. Error bars represent standard error mean (S.E.M.).

We first tested if an acute phase shift prior to training would alter acquisition of fear conditioned behavior (**Fig. 1A**). The control group (*n* = 8) was maintained on a 12∶12 LD cycle and is used for both the first and second experiment. The phase-shifted group of mice (*n* = 6) housed in 12∶12 LD was subjected to a 12 hr phase advance by extending the dark phase on the day prior to training (Day -1) resulting in an inversion of the lighting cycle on the day of training. On Day 0, both groups of mice were trained using the contextual fear conditioning protocol at their respective Zeitgeber Time (ZT) 3, which consists of 2 time-delayed pairings of a conditioned stimulus (CS, the context of the shock cage) and an unconditioned stimulus (US, foot shock) within a 6.5 min training session. There was no difference in acquisition of fear-conditioned freezing between the non-shifted and the phase-shifted groups (*t*-test for CS-US 1: *t*_12_ = 1.38, *P* = 0.19; CS-US 2: *t*_12_ = 0.37, *P* = 0.72), with both groups demonstrating 60 to 64% freezing by the second application of the CS-US (**Fig. 1B**). The mice were then tested for recall in 24 hr intervals on 7 subsequent days after training at ZT 3 (**Fig. 1C**). A two way repeated measures analysis of variance (2RM ANOVA) determined significant effects of the phase shift on recall of contextual fear conditioned freezing (*F*_1,12_ = 318.36, *P*<0.001) and between days (*F*_6,12_ = 62.40, *P*<0.001). Significant interaction was also determined for phase shift *x* day (*F*_6,91_ = 17.98, *P*<0.001). Post-hoc Bonferroni's *t*-test determined a significant reduction in recall in the phase shifted cohort (**Fig. 1C**).

Hence, an acute phase shift prior to training does not affect acquisition of fear-conditioned freezing, but has a negative effect on recall of contextual fear-conditioned freezing that persists over the testing period.

### Does an acute phase shift after training have an effect on recall?

**Figure 2:**
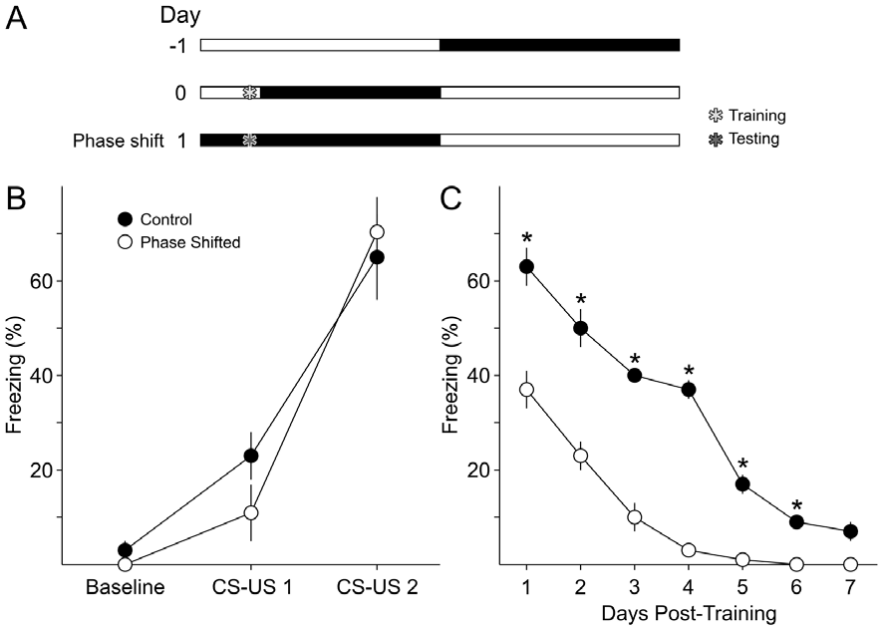
Acute phase shift after training reduced recall of contextual fear-conditioned behavior. **A**) Schematic illustration of the experimental design. On Day 0, two cohorts (*n* = 8 per group) were trained at ZT 3. After training, one cohort was immediately phase shifted while the other served as the control. 24 hr after training, both groups of mice were returned to the same context for testing once a day. **B**) Freezing in response to CS-US 1 and CS-US 2 was not different between the two cohorts of mice. **C**) Recall of the conditioned fear was significantly reduced by the phase shift.

Having determined that an acute phase shift prior to the training process does not affect acquisition, we tested the effects of an acute phase shift on recall after training (**Fig. 2A**). Two separate groups of mice (*n* = 8 per group) were trained at ZT 3 on Day 0. Acquisition of fear conditioned freezing was determined to be similar for both groups (**Fig. 2B**; *t*-tests CS-US 1: *t*_14_ = 0.96; *P* = 0.35; CS-US 2: *t*_14_ = −0.56, *P* = 0.56). The control group was maintained on the same 12∶12 LD cycle, and the second group was subjected to an immediate phase advance. Both groups of mice were returned to the conditioning chamber in 24 hr intervals post-training: ZT 3 for the control group and ZT 15 for the phase-shifted group. Testing of the phase-shifted group was performed under conditions of darkness at ZT 15. The phase shifted group performed considerably worse than the control group, demonstrating reduced fear conditioned freezing when placed in the same context (**Fig. 2C**). The adverse effect of the post-training phase shift was confirmed by a 2RM ANOVA, which determined a significant effect of phase shift (*F*_1,14_ = 220.47, *P*<0.001), between days (*F*_6,14_ = 82.26, *P*<0.001), as well as interaction between phase shift *x* day (*F*_6,111_ = 7.44, *P*<0.001). Post-hoc Bonferroni's *t*-tests revealed significantly reduced freezing in the phase shifted group on all 7 days of testing (**Fig. 2C**).

Thus, similar to our findings on phase shifts prior to training, subjecting mice to phase shifts immediately after the training event leads to markedly reduced contextual fear conditioned freezing when tested.

### Do acute phase advances versus phase delays have different effects on recall?

**Figure 3:**
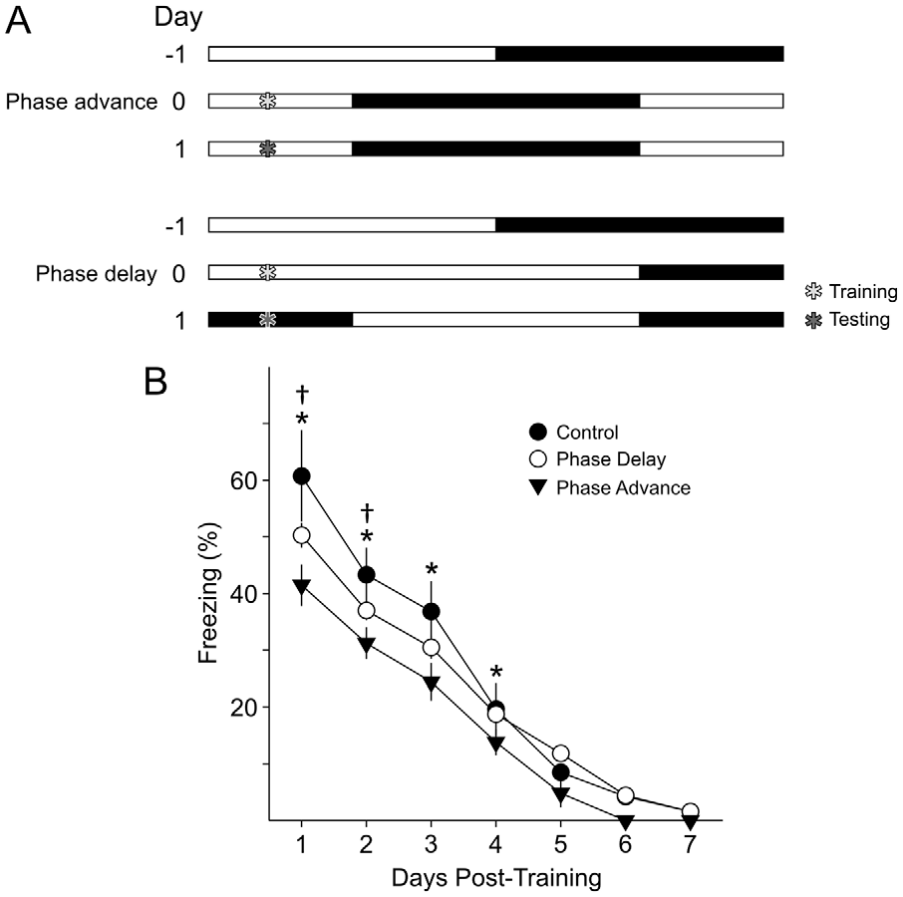
Both phase advances and delays of the LD cycle reduced recall of contextual fear-conditioned behavior. **A**) Schematic illustration of the experimental design. On Day 0, three cohorts (*n* = 6 per group) were trained at ZT 3. Acquisition of fear conditioned freezing behavior was not significantly different between the three groups (data not shown). After training, one cohort was subjected to a 6 hr phase advance of the LD cycle, a second cohort was subjected to a 6 hr phase delay of the LD cycle and the third cohort was kept under the same LD cycle as controls. All cohorts were tested for recall in 24 hr intervals. **B**) Recall of the conditioned fear was significantly reduced by both the phase advance and delay of the LD cycle. 2RM ANOVA was performed with post-hoc Bonferroni's *t*-tests, and “*” indicates significant differences between the control and phase advanced cohort while the “†” symbol indicates significant differences between the control and phase delayed cohort.

Having determined that acute phase shifts prior to as well as after training specifically affect recall, we wished to determine if the direction of the phase shift had different effects on the recall of fear-conditioned behavior (**Fig. 3A** **)**. We trained 3 separate cohorts of mice (*n* = 6–7 per group) and subjected one cohort to a 6 hr phase advance after training, testing this phase advanced cohort 24 hr post-training at their new ZT 9. The second cohort was subjected to a 6 hr phase delay after training, and tested 24 hr post-training at the new ZT 21 in the dark. The third cohort was not phase shifted. Acquisition was not different between the three groups (one way ANOVA; CS-US 1: *F*_2,18_ = 2.74, *P* = 0.09; CS-US 2: *F*_2,18_ = 3.04, *P* = 0.07). In contrast, retention of the contextual fear conditioned behavior was again found to be significantly different between the phase shifted groups by 2RM ANOVA (**Fig. 3B**; *F*_2,17_ = 9.32, *P* = 0.002) with significant differences between days (*F*_6,17_ = 209.79, *P*<0.001) and significant interaction between phase shift *x* day (*F*_12,139_ = 3.62; *P*<0.001).

Post-hoc Bonferroni's *t*-tests showed that both the phase advanced and phase delayed groups had significantly reduced recall of contextual fear conditioned behavior on the first day of testing compared to the un-shifted group (Advance: *t*_12_ = 6.36, *P*<0.001; Delay: *t*_12_ = 4.12, *P*<0.001) but could not detect significant differences between the phase advanced versus phase delayed group (*t*_13_ = 2.34, *P* = 0.07) as well as on all subsequent days of testing. Both the phase advanced and phase delayed groups continued to show significantly lower recall of contextual fear conditioned behavior on day 2 (Advance: *t*_12_ = 4.12, *P*<0.001; Delay: *t*_12_ = 2.65, *P* = 0.03). By day 3, the conditioned behavior was no longer different between the phase delayed group and control (Advance: *t*_12_ = 3.46, *P* = 0.003; Delay: *t*_12_ = 1.92, *P* = 0.18).

Both acute phase advances and delays lead to impairments in recall of contextual fear conditioned behavior.

### Does the magnitude of the acute phase shift have varying effects on recall?

**Figure 4:**
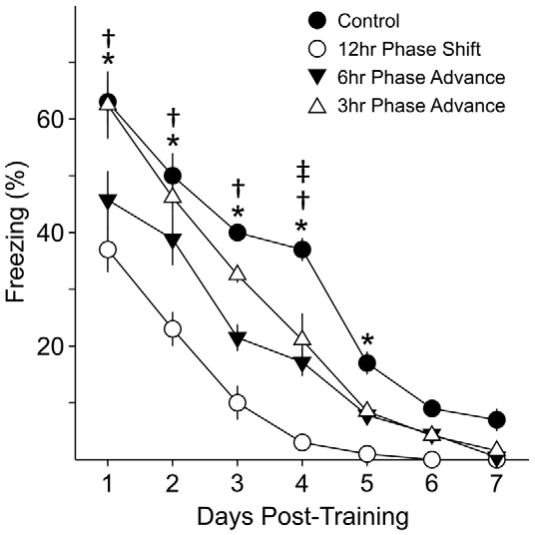
Phase advances of 6 hrs or more reduced recall of contextual fear-conditioned behavior. On Day 0, four cohorts (*n* = 6–8 per group) were trained at ZT 3. Acquisition of fear conditioned freezing behavior was not significantly different between the groups (data not shown). After training, one cohort was subjected to a 12 hr phase shift, a second cohort was subjected to a 6 hr phase advance and the third cohort subjected to a 3 hr phase advance. All cohorts of mice were tested for recall of contextual fear conditioned behavior in 24 hr intervals. Recall of the conditioned fear was significantly reduced by the 12 and 6 hr phase advance of the LD cycle. 2RM ANOVA was performed with post-hoc Bonferroni's *t*-tests, and “*” indicates significant differences between the 12 hr phase shifted group and control; the “†” symbol indicates significant differences between the 6 hr phase advanced group and control; “‡” indicates significant differences between the 3 hr phase advanced group and control.

We wished to determine the minimum magnitude of phase shift that would produce an effect on recall of contextual fear (**Fig. 4**). We subjected 3 separate cohorts to phase advances of varying degrees (3, 6 and 12 hr) after a training session at ZT 3 and compared these to a fourth cohort that was not phase shifted. Acquisition of fear conditioned freezing was not different between the 4 groups (one way ANOVA; CS-US 1: *F*_3,26_ = 1.56, *P* = 0.22; CS-US-2: *F*_3,26_ = 0.30, *P* = 0.82). Testing for fear conditioned behavior was performed for all 4 groups at 24 hr intervals post-training. 2RM ANOVA determined significant differences between phase shifted groups (*F*_3,26_ = 49.67, *P*<0.001) and between days (*F*_6,26_ = 181.51, *P*<0.001), with significant interaction between phase shifts *x* day (*F*_18,209_ = 3.78, *P*<0.001).

Both the 12 hr and 6 hr phase-advanced groups displayed significantly reduced recall compared to the un-shifted control group on the first day of testing (12 hr shift: *t*_15_ = 6.62, *P*<0.001; 6 hr advance: *t*_14_ = 4.28, *P*<0.001). The recall of contextual fear conditioned freezing of the 3 hr phase advanced group was not significantly different from control (3 hr advance: *t*_14_ = 0.20, *P* = 1.00). Curiously, differences were observed between the 3 hr phase advance group and the control group on only day 4 of testing (*t*_14_ = 3.93, *P*<0.001). On the first test, no difference was observed between the 12 hr shifted cohort and the 6 hr phase advanced cohort (*t*_14_ = 2.11, *P* = 0.22), but further tests on days 2 and 3 showed reduced freezing in the 12 hr shift cohort compared to the 6 hr phase advance cohort (day 2: *t*_14_ = 3.86, *P*<0.001; day 3: *t*_14_ = 2.67, *P*<0.001).

Thus, the increasing magnitudes of phase shift cause greater impairment of recall of contextual fear conditioning, with the 12 hr phase inversion causing the most disruption, followed by the 6 hr phase advance, with minimal effects observed after a 3 hr phase advance.

### Does peak recall change according to the new lighting schedule?

**Figure 5:**
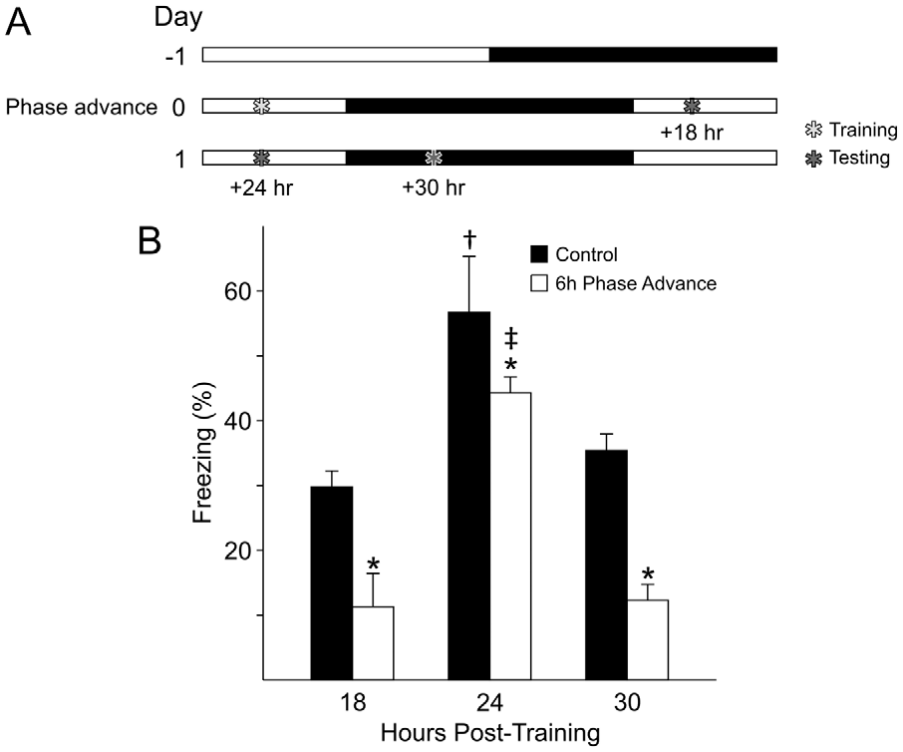
The peak recall of conditioned fear behavior occurred 24 hrs after training with or without a phase shift. **A**) Schematic illustration of the experimental design. On Day 0, six cohorts (*n* = 6 per group) were trained at ZT 3. Acquisition of fear conditioned freezing behavior was not significantly different between the six groups (data not shown). After training, three cohorts were subjected to a 6 hr phase advance of the LD cycle. Cohorts, one phase shifted and one control group were tested for recall of contextual fear-conditioned behavior at 18, 24 or 30 hrs intervals after training. **B**) Recall of the conditioned fear was reduced by the phase advance of the LD cycle at each of the three intervals tested. The peak recall for both groups was found 24 hrs after training. 2RM ANOVA was performed with post-hoc Bonferroni's *t*-tests, and “*” indicates significant differences between the phase shifted and control groups. Within groups (control and phase advanced), one way ANOVA revealed differences between testing times (18, 24, 30 hrs post training) with the interval of peak recall in the control group indicated with a “†”, and within the phase advanced group as indicated by a “‡”.

A critical control experiment was to determine if the peak of recall following a phase shift was also shifted by the change in the LD cycle (**Fig. 5A**). In many cases, peak recall is observed in 24 hr intervals after training [38]. Following a 6 hr phase advance, we tested separate cohorts of mice at 18, 24 and 30 hr after training at ZT 3 to account for any possible shift in the peak recall. 6 separate cohorts of mice were trained at ZT 3 and each cohort was tested only once at 18 hr, 24 hr or 30 hr post-training time. 3 cohorts were left un-shifted as controls to be tested at ZT 21 (18 h post-training), ZT 3 (24 hr post-training) and ZT 9 (30 hr post-training). 3 cohorts were subjected to a 6 hr phase advance following training, and tested at the new ZT 3 (18 hr post-training), ZT 9 (24 hr post-training) and ZT 15 (30 hr post-training).

There was equal acquisition across all 6 groups (one way ANOVA; CS-US 1: *F*_5,24_ = 1.02, *P* = 0.432; CS-US 2: *F*_5,24_ = 0.58, *P* = 0.72). Comparison of the recall of fear conditioned behavior at all times of testing by one way ANOVA revealed significant differences between testing times (*F*_5,24_ = 51.45, *P* <0.001; **Fig. 5B**). Post-hoc Bonferroni's *t*-tests confirmed that the control group's recall at 24 hr was significantly higher than recall at 18 hr and 30 hr post-training (control 24 hr vs. 18 hr: *t*_6_ = 10.94, *P*<0.001; 24 vs. 30 hr: *t*_6_ = 9.14, *P*<0.001) and was also significantly greater than all times of testing of the phase-shifted groups (control 24 hr vs. shifted 18 hr: *t*_8_ = 13.30, *P*<0.001; control 24 hr vs. shifted 24 hr: *t*_8_ = 4.97, *P*<0.001; control 24 hr vs. shifted 30 hr: *t*_7_ = 12.47, *P*<0.001). Within the shifted group, the recall of contextual fear conditioning peaked in the group tested at 24 hr post-training (shifted 24 hr vs. 18 hr: *t*_8_ = 8.39, *P*<0.001; 24 hr vs. 30 hr: *t*_7_ = 7.67, *P*<0.001).

Within testing times, the phase shifted group consistently demonstrated reduced recall of contextual fear conditioned behavior than the un-shifted groups (18 hr: *t*_8_ = 5.09, *P*<0.001; 24 hr: *t*_7_ = 4.97, *P*<0.001; 30 hr: *t*_7_ = 6.03, *P*<0.001; **Fig. 5B**). Recall in the phase shifted group at 24 hr post-training was greater than the non-shifted group at 18 hr post-training (*t*_7_ = 3.99, *P* = 0.01), but otherwise, recall exhibited by the phase-shifted groups did not exceed that of the non-shifted groups at other testing times.

The results from these experiments suggest that the 24 hr interval post-training remains the time of highest recall regardless of the new zeitgeber time.

### Does the acute phase shift lead to an altered fear response?

**Figure 6:**
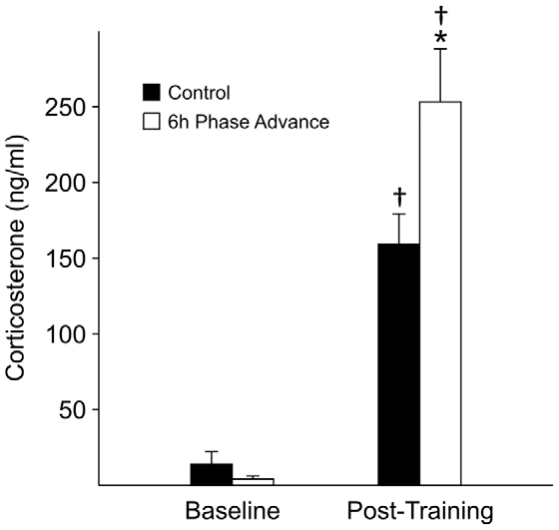
Phase advance of the LD cycle enhanced the magnitude of the stress-evoked corticosterone response. Four cohorts (*n* = 6–8 per group) of adult male mice were entrained to a 12∶12 LD cycle for at least 2 weeks. On Day -1, two cohorts were subjected to a 6 hr phase shift of the LD cycle. On Day 0, serum corticosterone levels were measured at ZT 3. Baseline concentration of serum corticosterone was not different between the control and phase shifted groups. The cohorts that underwent training for fear conditioning exhibited significant increases in corticosterone. The magnitude of this stress-evoked response was significantly increased in the phase-shifted group. 2RM ANOVA was performed with post-hoc Bonferroni's *t*-tests and “*” indicates significant differences between training evoked corticosterone responses, while “†” indicates significant differences between baseline and trained mice.

Corticosterone is a hormone secreted with a robust circadian rhythm whose levels can regulate many aspects of learning and memory (e.g., [39]). To determine if acute phase shifts have an impact on baseline circulating corticosterone, we sampled from control mice and mice that had been subjected to a 6 hr phase advance on the day prior to sampling (**Fig. 6**). No significant differences were measured in the serum corticosterone levels at ZT 3 between the un-shifted and phase-shifted groups (*t*_6_ = 1.45, *P* = 0.21). To determine if the corticosterone response to the training protocol was altered, we obtained blood samples from mice 20 min after the 2 CS-US training procedure from a non-shifted group and a group that had been subjected to a 6 hr phase advance on the day prior to training. Circulating corticosterone was significantly increased from baseline levels in both groups of mice, and the serum concentration of corticosterone was significantly increased in the phase-shifted mice (*t*_9_ = −2.45, *P* = 0.04) compared to non-shifted controls. Two way ANOVA confirmed a significant training-evoked corticosterone response (*F*_1,16_ = 70.49, *P*<0.001) as well as a significant interaction between the training procedure and phase shift (*F*_1,16_ = 4.98, *P* = 0.04).

Thus, the rapid shift of the LD cycle alters the stress-evoked corticosterone response in the mice.

### Does the acute phase shift cause sleep deprivation?

Several lines of evidence suggest that sleep plays some type of critical role in memory consolidation and many studies have found evidence that sleep deprivation interferes with the recall of learned behaviors (e.g., [40]). To determine if the phase shifting procedure interferes with sleep in the mice, we performed electroencephalogram (EEG) and electromyogram (EMG) recordings to determine the total amount of sleep in mice 24 hour prior to and after a 6 hr phase advance (**Table 1**). We found that the percentage of time spent awake was not significantly different before or after the phase advance (*t*_9_ = −0.24, *P* = 0.82). There was also no significant change in the amount of non-rapid eye movement (NREM) and REM sleep (NREM: *t*_9_ = 0.22, *P* = 0.83; REM: *t*_9_ = 0.41, *P* = 0.69).

**Table 1:** Percentage of time spent awake and in NREM and REM sleep as determined by EEG/EMG recordings from mice 24 h before and after an acute 6 hr phase advance of an LD cycle.

	Baseline	Phase Advance
Waking	51.3±3.2%	53.0±7.8%
NREM	42.7±2.6%	41.4±6.7%
REM	6.0±0.2%	5.6±1.3%

No significant differences were found between the baseline and post-phase shift amounts of wake and sleep (REM and NREM sleep).

**Figure 7:**
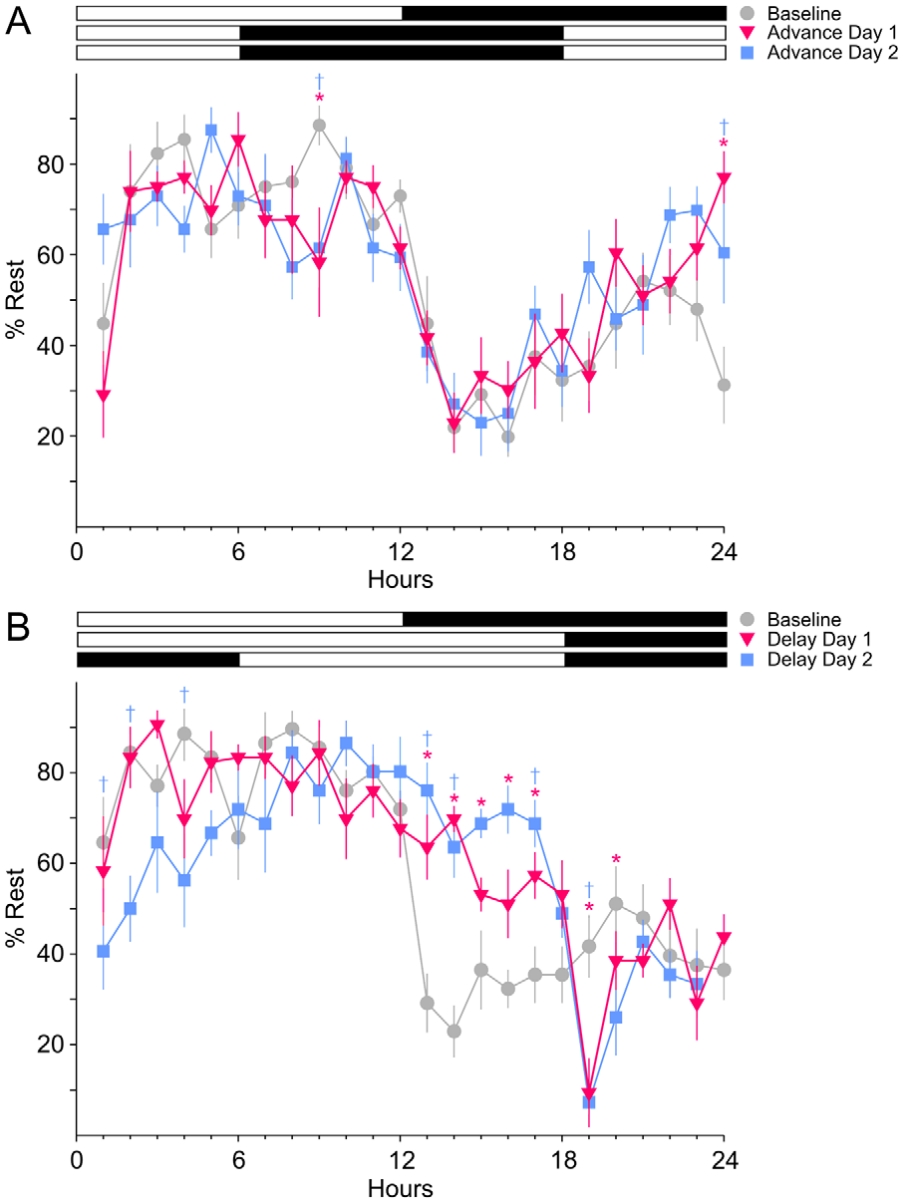
Phase advance of the LD cycle alters the distribution but not the total amount of sleep. A single cohort (*n* = 8) of adult male mice were entrained to a 12∶12 LD cycle for 2 weeks. The mice were videotaped for 24 hrs to establish a baseline and an additional 48 hr during which the mice were subjected to a 6 hr phase shift of the LD cycle. The video was scored every 5 min to determine if the mice were awake or asleep. In both **A** and **B**, the significant differences between baseline and the first day of the phase shift as determined by 2RM ANOVA with post-hoc Bonferroni's *t*-tests are denoted by “*” and the significant differences between baseline and the second day of the phase shift by “†”. **A**) A 6 h phase advance of the LD cycle did not change the total amount of sleep over a 24 h cycle compared to the baseline recordings, but resulted in minor changes in the distribution of sleep. **B**) A 6 h phase delay of the LD cycle resulted in increased sleep during and after the phase shift, and caused an immediate increase in sleep during the extended 6 h of light, as well as a corresponding decrease in sleep during the dark hours of the following day.

To examine possible changes in the distribution of sleep, we recorded rest/wake behavior from mice subjected to acute phase shifts. Video recordings of the sleep/wake behavior were made and visually scored (*n* = 8) before and after a 6 hr phase advance in the LD cycle. Our behavioral data confirmed our EEG findings that the amount of sleep within a 24 hr interval does not change before (55.5±4.4% rest/24 hrs) or after (56.8±3.8% rest/24 hrs) an acute phase advance of the LD cycle (2-way ANOVA: *F*_2,7_ = 0.81, *P* = 0.47). However, there was some evidence that the temporal distribution of sleep was altered following the phase advance (day *x* hour interaction: *F*_46,7_ = 2.22, *P*<0.001; **Fig. 7A**). Next, we examined the impact of a 6 hr phase delay. In this case, there was a small but significant increase in the amount of sleep (baseline: 58.3±4.7% rest/24 hrs) after (61.8±4.1% rest/24 hrs) the phase shift (2RM ANOVA: *F*_2,7_ = 4.17, *P* = 0.021). In addition, there was evidence that the temporal distribution of sleep was altered following the phase delay (day *x* hour interaction: *F*_46,7_ = 4.87, *P*<0.001; **Fig. 7B**).

These data demonstrate that the acute phase shift (6 hrs) did not result in sleep deprivation as measured over a 24 hr period. A phase delay (6 hrs) may have actually increased sleep during this time interval (24 hrs). The temporal distribution of rest was altered by the shift in the LD cycle.

### The effect of prior experience of phase shifts on recall

**Figure 8:**
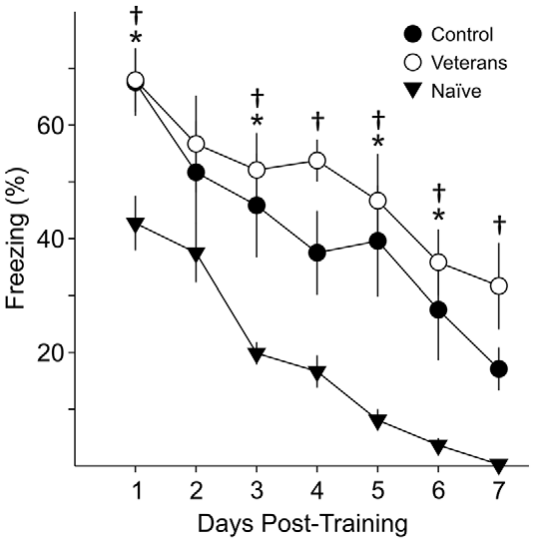
Prior experience with phase shifts reverses the impact of the jet lag on the recall of conditioned fear behavior. One cohort of mice (*n* = 8) was subjected to 3 successive combinations of phase advances and delays spaced out on a weekly basis (veterans), and compared to mice that were left un-shifted (control, n = 8) as well as a third cohort of mice that were subjected to an acute 6 hr phase advance after training (naïve, n = 8). 2RM ANOVA was performed with post-hoc Bonferroni's *t*-tests and “*” indicates significant differences between control and naïve cohorts and “†” indicates significant differences between the veterans and naïve cohorts. No statistical differences were found between the un-shifted control group and the phase-shifted veterans group on all 7 days of testing.

In this experiment, we examined the effect of prior exposure to phase shifts on recall of contextual fear-conditioning (**Fig. 8**). One cohort of mice was subjected to repeated phase shifts on a weekly basis. The LD cycle was phase advanced by 6 hr, followed by a 6 hr delay after a week. This was repeated twice prior to training to produce a cohort of mice that were considered “Veterans” of phase shifts. Acquisition of fear conditioned freezing was not altered by the multiple phase shifts prior to training (one way ANOVA: CS-US 1: *F*_2,17_ = 2.12, *P* = 0.15; CS-US 2: *F*_2,17_ = 0.37, *P* = 0.69). Immediately following training, the veteran cohort was phase-shifted along with trained mice that were naïve to phase shifts. A third set of mice was left un-shifted as controls. Comparison of recall of contextual fear conditioned behavior of all three groups by 2RM ANOVA revealed a significant effect of phase shift (*F*_2,15_ = 12.83, *P*<0.001) and day (*F*_6,15_ = 28.47, *P*<0.001), but no interaction between shift and day (*F*_12, 125_ = 0.79, *P* = 0.66). Consistent with previous experiments, the naïve phase-shifted group displayed lower levels of recall compared to the control non-shifted group on the first day of testing (post-hoc Bonferroni's *t*-test: *t*_11_ = 2.79, *P* = 0.02). Surprisingly, the veterans of previous phase-shifts did not display deficits in recall following a phase shift when compared to controls (*t*_11_ = 0.05, *P*>0.99) and had significantly better recall than the mice naïve to phase shifts (*t*_11_ = 2.83, *P* = 0.02). Significant differences on subsequent days are indicated in Fig 8.

Pre-exposing the mice to multiple phase shifts before training can ameliorate the detrimental effects of an acute phase shift on recall of contextual fear conditioned behavior.

## Discussion

In this study, we found that acute phase shifts selectively affect recall of the hippocampal-dependent contextual fear conditioned behavior, regardless of whether we phase shifted the mice before or after training. Our findings are consistent with previous studies on phase shifts and cognition [33], [34], but one critical difference is that we applied the phase shift only once to mice that were naïve to such manipulations. Hence, we were able to demonstrate that a rapid shift in the lighting cycle produces a dramatic reduction in recall without a significant effect on acquisition (**Fig. 1** **&** **2**). The duration and severity of jet lag depends on the number of time zones crossed. For the circadian system, the larger the phase shift, the longer the duration required for re-synchronization to the new lighting schedule. For example, several studies suggest that the circadian system would require more than 6 days to recover from the 6 hr phase advance used in the present study [3], [41], [42], [43]. We were able to demonstrate that the larger the phase shift, the larger the impact on recall (**Fig. 4**) with even a 3 hr phase advance having some impact on recall compared to untreated controls. Using this same behavioral assay, we previously found that a mutation in one of the key clock genes (*Period2*) as well as the loss of vasoactive intestinal peptide, a signaling molecule critical for coupling within the central clock, reduced recall, but not acquisition, of conditioned fear [8], [26]. Collectively, our findings are consistent with a role for the circadian system in the consolidation of memory.

Several lines of evidence indicate that phase advances of the LD cycle are more disruptive than phase delays. In general, an organism's behavioral activity-rest cycle can re-synchronize to a phase delay of the LD cycle rapidly while synchronization to a phase advance is much more gradual. For example, in mice, re-synchronization to a 6 hr phase delay occurs within a couple of days, while re-synchronization to a 6 hr phase advance may take 5–6 days [42]. In older mice, repeated phase advances can increase mortality, an effect not seen with phase delays [44]. These studies suggest that phase advances may be more disruptive to cognitive processes than phase delays. In the present study (**Fig. 3**), we found that both advances and delays disrupted the recall of the conditioned fear. The impact of the phase advance was larger than the phase delay at all time points tested, so it is possible that future work will find more robust differences. Earlier work with rats also found that both advances and delays of the LD cycle disrupted memory [34]. Perhaps the difference between advances and delays on cognitive processes lies more in the duration of the disruption than its magnitude.

One downside of using the 12 hr phase shift (**Fig. 1** **&** **2**) as a drastic disruption of the circadian system is the possibility that the re-entrainment could take place via phase advances or delays. Our series of different durations of phase shifts described in **Fig. 4** confirmed that although the 12 hr phase shift has the most disruptive effect on memory, 6 and 3 hr shifts also have a significant impact on recall of fear-conditioned behavior, and the 6 hr protocol was hence used in all subsequent mechanistic experiments to allow interpretation of the effects of direction of shift and other factors that could affect memory. A further possible confound of the post-training phase shift experiment described in **Fig. 2** is the difference in the lighting conditions between training (light phase) and testing (dark phase post-shift). We have previously shown that recall is higher in the day than in the night [13]. While we cannot rule out some direct effect of dark reducing recall in this one experiment, most of our experiments were carried out with shorter phase shifts in which both training and testing were carried out under the same lighting conditions that continued to affect recall (e.g. **Fig. 4** **&** **5**). Furthermore, the experiment described in **Fig. 5** shows no difference in freezing between the non-shifted animals tested at non-24 hr intervals in the dark (18 hr) and light (30 hr) phase. Similarly, the mice subjected to a phase shift prior to testing do not show a difference in freezing between the non-24 hr interval testing phases in the light (18 hr) and dark (30 hr). For these reasons, we do not feel that the acute effects of lighting conditions were an interpretational problem for these studies.

### Peak performance still occurs 24 hrs after training in the phase shifted group

Behaviorally, there is a long history of work demonstrating that peak performance in the recall of a number of behavioral tasks varies with time of day [9]–[12] and circadian time [13], [14], [15]. This type of research has led in most behavioral learning protocols to keep the interval between training and testing at 24 hrs. This prior work also raises the possibility that the 6 hr advance in the LD cycle induced an immediate 6 hour shift in the peak of recall. If this were the case, then the peak of recall would be 18 hrs after training in the phase advanced group while remaining at 24 hrs after training in the control group. We examined this possibility by training mice that were on a stable LD cycle and then testing them at 18, 24, and 30 hrs after training (**Fig. 5**). The control mice showed a clear peak of recall of training 24 hrs after training, confirming prior work. Interestingly, the phase advanced cohort also showed a peak in recall 24 hrs after training. The 6 hr advance did not shift the peak in performance to 18 hrs after training. Therefore, the “time-stamp” of 24 hr for peak recall was not affected by phase shifts, and confirmed that the reduced recall we observed after a phase shift is not due to a shift in the timing of the peak recall.

### The jet lag protocol alters the magnitude of the stress response but not baseline levels of corticosterone

Stress and the release of corticosterone is an important modulator of learning and memory [39], [45], [46]. With contextual fear conditioning, increasing corticosterone can facilitate consolidation [47], [48], [49] or interfere with recall [50], [51], [52]. Corticosterone is a hormone secreted with a robust circadian rhythm, with peak secretion during the late day, ∼ ZT 10, in nocturnal rodents [53]. Anatomical studies have provided evidence that the paraventricular nucleus (PVN) receives innervations from the SCN. Release of corticotrophin releasing factor by neurons within the PVN is the critical step in stimulating adrenocorticotropic hormone (ACTH) release from the pituitary and thus the activation of the hypothalamic-pituitary-adrenal axis [54]. SCN-lesioned rats show a loss of daily rhythm in ACTH and corticosterone [55], [56], [57]. In this study, we determined the impact of the 6 hr phase advance on the levels of corticosterone in the mice. While we only sampled at one time of day, we did not see evidence that unstimulated, baseline levels were increased by the phase shift (**Fig. 6**). In contrast, the stress (foot shock) evoked responses were significantly larger in the phase shifted group. So it is possible that higher corticosterone levels during recall played a role in the reduced memory in the phase shifted groups. In flight crews who habitually experience travel between more distant time zones, there is evidence for both higher salivary cortisol and reduced performance of vigilance tasks [31], [58].

### The jet lag protocol alters the temporal distribution but not total amounts of sleep

Sleep immediately after a training session has been shown to be critical for consolidation of contextual fear conditioned memory [59], [60] as well as many other learned behaviors [40], [61]. In humans, sleep disturbances are a common complaint after jet travel crossing a number of time zones [62], [63]. To examine the possibility that the 6 hr phase advance caused sleep deprivation in mice, we examined pre- and post-phase shift sleep/wake patterns using EEG recording in freely moving mice. We found no significant differences in the amount of NREM or REM sleep before and after the phase advance (**Table 1**). Surprisingly, we could not find other studies that had examined the impact of experimental jet lag on sleep in mice. In rats, there has been one report that phase advances of the LD cycle led to an increase in NREM and REM sleep [64]. Our phase advance protocol results in one shorted day, and it has been shown that rats and hamsters housed under short photoperiod (8∶16 LD) show altered sleep patterns but the short photoperiod does not affect sleep homeostasis [65], [66]. To further explore the sleep/wake patterns, we turned to behavioral measures of sleep [67], [68]. We measured the patterns of sleep/wake before and after a 6 hr phase advance. The results (**Fig. 8**) clearly show a change in the temporal distribution of sleep but do not show an overall loss of sleep. Thus the impact of jet lag on recall occurred without producing sleep deprivation. Future studies will need to explore the relationship between misalignment of sleep on memory consolidation.

### Experience can reduce the impact of jet lag on the conditioned fear

As a final experiment, we tried to further disrupt the circadian system by subjecting the mice to repeated phase shifts, but discovered that prior experience of phase shifts appears to ameliorate the adverse effects on recall. This observation may explain some apparent contradictions in the literature. A previous study by Craig and McDonald showed that chronic or serial jetlag in rats impairs acquisition, but in contrast to our findings, chronically and acutely jetlagged rats did not appear to show deficits in recall of contextual fear conditioned freezing [69]. This discrepancy could be due to differences in application of “acute” phase shifts. In our study, all phase shifts were acutely applied to mice naïve to phase shifts, whereas Craig and McDonald applied serial phase shifts over several days to produce their acute jetlag model. In fact, their study agrees with our finding that multiple serial exposures to phase shifts can compensate for the negative effects of acute phase shifts on memory. The data suggest that it is possible to design treatments that can reduce the cognitive impact of circadian de-synchronization.

### Phase shifts desynchronize the network of circadian oscillators: mechanisms

Previous studies have shown that when rodents are subjected to acute phase shifts of the LD cycle, de-synchrony results within core clock genes within the SCN [42], between different regions within the SCN [35], [36], [70] and between the SCN and peripheral oscillators [71]. Within circuits involved in learning and memory, it has been demonstrated that the amygdala takes longer to re-entrain to phase shifts of the LD cycle than the SCN [72], [73]. Nuclei within the amygdala (central and basolateral) and as well as the dentate gyrus region of the hippocampus exhibit rhythms in gene expression which are dependent on an intact SCN [5]. The hippocampus also exhibits rhythms in clock gene expression [7], [8], [26] that are independent of the SCN [8]. By applying an acute phase shift, we are most likely uncoupling the tightly synchronized network of circadian oscillators, including regions of the brain responsible for learning and memory. We speculate that this disruption in the coordination of clock gene expression within different neural structures lies at the heart of memory deficits.

Consolidation of memory involves changes in gene expression [74], [75] and is prevented by inhibitors of transcription and translation. The molecular circadian clock regulates the temporal pattern of transcription and we believe that by this mechanism, disruptions in the molecular clock could also disrupt consolidation of memory. Previous work has also found evidence that levels of adenylyl cyclase 1 expression [76] as well as cAMP and MAPK activity in the hippocampus [17] exhibit daily oscillations. Previous work in *Aplysia* implicates the circadian gating of the MAPK pathway as the mechanistic control point for circadian regulation of sensitization [16]. These results also raise the possibility that jet lag evoked disruptions in intracellular signaling pathways may be an important part of the observed deficits in recall.

### Conclusions and Significance

In the present study, we demonstrate that single acute phase shifts can reduce recall of a learned behavior, presumably through altering memory consolidation. Among other novel findings, we demonstrate that the 24-hr interval between training and testing still produces the strongest recall even in phase shifted mice. We were able to disassociate the impact of the circadian disruption from the total amount of sleep as the mice were not sleep deprived. The temporal distribution of sleep was disrupted and future studies will need to explore the importance of when sleep occurs on memory consolidation. Our data adds to a body of studies that have shown that a functioning circadian system is important for long-term memory. Memory deficits have been found in several lines of mice with mutations impacting the generation of robust circadian rhythms in behavior [8], [21]–[26]. Similarly, environmental manipulations, including chronic phase shifts of the LD cycle, that disrupt circadian rhythms without genetic mutations also disrupt memory in different tasks [28]–[34], [69], [77]. We think that the broader hypothesis that internal desychronization of a network of circadian oscillators results in memory deficits is clinically important. Patients with a variety of psychiatric and neurological disorders exhibit disruptions in their sleep and circadian rhythms. If our hypothesis is correct, these circadian disruptions may contribute to the cognitive symptoms experienced by a range of patients.

## Methods

### Experimental animals

Two to four-month old C57Bl/6 male mice were used in this study. All mice were housed in cages within light-tight chambers with controlled lighting conditions. The experimental protocols used in this study were approved by the UCLA Animal Research Committee (ARC 1998-183-41), and all recommendations for animal use and welfare, as dictated by the UCLA Division of Laboratory Animals and the guidelines from the National Institutes of Health, were followed.

### Experimental lighting conditions

In all experiments, mice were entrained to a 12∶12 LD cycle for at least one week prior to the start of all experiments (light intensity 36 µW/cm^2^≅120 lux). All times are reported as Zeitgeber Time (ZT), where ZT 0 corresponds to the start of the light period, and ZT 12 refers to the start of the dark period. Training procedures were done during the day at either ZT 3 (early day) or ZT 6 (mid-day). All testing procedures were carried out in intervals of 24 hr based on the initial training time unless otherwise stated. Acute changes to the lighting schedule were performed relative to the lighting cycle for each independently controlled chamber.

### Training and testing procedure

Contextual fear conditioning was performed using previously published protocols [13], [26]. Briefly, on the day of training (denoted as Day 0), mice were placed individually into cages and allowed to acclimatize to the new environment (conditioned stimulus; CS) for 3 min after which time animals received a 2 sec 0.2 mA foot shock (unconditioned stimulus; US). The training protocol consisted of 2 of these conditioned and unconditioned stimulus (CS-US 1 and 2) pairings with an inter-trial interval of 64 sec. At the end of the last CS-US pairing, the mice were left in the cage for a further 64 sec, after which they were returned to the home cages. On the day of testing (Day 1 to 7), mice were placed individually into the same conditioning chamber for 6 min. The fear conditioned freezing behavior was scored as previously described [13], [26]. When tested in the dark, handling of the mice was performed using an IR viewer (FJW Industries, Ohio) and recording of fear conditioned behavior was done using an IR-capable camcorder (Sony, DRC-DVD408, NY).

### Corticosterone measurements

Circulating corticosterone concentration in serum was determined as previously described [78]. Briefly, trunk blood was collected from mice anesthetized with isoflurane. The serum supernatant obtained by centrifugation of clotted blood at 1000× *g* was assayed by competitive enzyme immunoassay (Correlate-EIA Corticosterone, Assay Designs, Ann Arbor, MI). The intra-assay CV was <8%, the inter-assay CV was <13.1% and the sensitivity was 27 pg/ml.

### Sleep measurements

EEG and EMG recordings and vigilance state scoring were performed as described previously [79]. EEG recordings before and after the phase shift were performed on the same mice. Vigilance state values were averaged to reflect the 24 hr levels of time spent awake and in NREM and REM sleep. Behavioral measurements of sleep were performed using surveillance camera system (Gadspot, GS-335C, CA). The same cohort was used for baseline and post-phase shift measurements. Mice were visually scored for sleep/wake activity in 5 min intervals. These values were summed and hourly percentages of sleep/wake were determined. The sleep state is marked by several easily observed behaviors, including adoption of a species-specific sleep posture with the eyes closed [67]. Thus, we scored an animal as having been behaviorally asleep only if its eyes were closed as it either lay on its side or sat curled up with the head tucked into the body and if it made no movement other than very slight and brief transitional changes in posture. This type of strategy has been previously used to assess the basic temporal distribution of behavioral sleep across a 24-h period (e.g., [68]).

### Statistical analysis

All reported values are mean ± SEM unless otherwise stated. To make simple comparisons between groups, Student's *t*-tests were used. In the cases in which repeated measurements were made from single animals, the data was analyzed using a two-way repeated measure (2RM) analysis of variance (ANOVA) followed by Bonferroni's *t*-tests for multiple comparisons. For all tests, values were considered significantly different at *P*<0.05. To compare recall for animals tested once at 18-, 24- or 30-hrs following training, one-way ANOVA was used followed by Bonferroni's post-hoc *t*-tests for pair-wise comparisons. One-way ANOVA with Bonferroni's post-hoc *t*-test was also used to test recall for vets vs. naïve, advances vs. delays, and 12 vs. 6 vs. 3 hr shifts.
